# Balloon septostomy: A novel approach for crossing a double interatrial septum during pulsed field ablation

**DOI:** 10.1016/j.hrcr.2024.05.001

**Published:** 2024-05-09

**Authors:** Joseph J. Souza, Raymond K. Nelson, Christopher C. Reynolds, Rahul Dhawan, Hae W. Lim, Michael G. Antimisiaris

**Affiliations:** ∗ Heart & Vascular Institute of Eastern Kentucky at Pikeville Medical Center, Pikeville, Kentucky; † Medtronic, Inc, Minneapolis, Minnesota

**Keywords:** Atrial fibrillation, Pulsed field ablation, Double interatrial septum, Balloon septostomy, Transseptal

**Key Teaching Points**

• Double interatrial septum is a rare anatomic anomaly.
• Transseptal puncture and access may be difficult in the setting of double interatrial septum.
• Safe transseptal access may be achieved using balloon septostomy.

## Introduction

Catheter ablation is a well-established treatment option for patients with symptomatic atrial fibrillation (AF),^1^ and procedural success is predicated on successful transseptal puncture and easy access to the left atrium. In fact, when transiting across the atrial septum, there are specific locations of preferred puncture site (eg, a low and anterior approach) that can convey a mechanical advantage for the placement of the ablation catheter (and delivery sheath) at anatomical locations, including the right-sided pulmonary veins.^2^ While rare anatomic cardiac anomalies of the atrial septum are known and described,^3^, ^4^, ^5^ these anatomic anomalies can create challenges during the ablation procedure. There have been a few reports of transseptal puncture of double atrial septum using traditional thermal ablation catheters^6^^,^^7^; however, there are no reports of a double septum crossing while using a pulsed field ablation (PFA) catheter. Herein, we describe a patient with a double interatrial septum that required a novel crossing technique to facilitate an ultimately successful catheter ablation of AF using a novel PFA catheter. Specifically, a balloon-facilitated septostomy was used to transit the double interatrial septum to facilitate sheath entry into the left atrium to support an AF catheter ablation via PFA device.

## Case report

**Figure 1:**
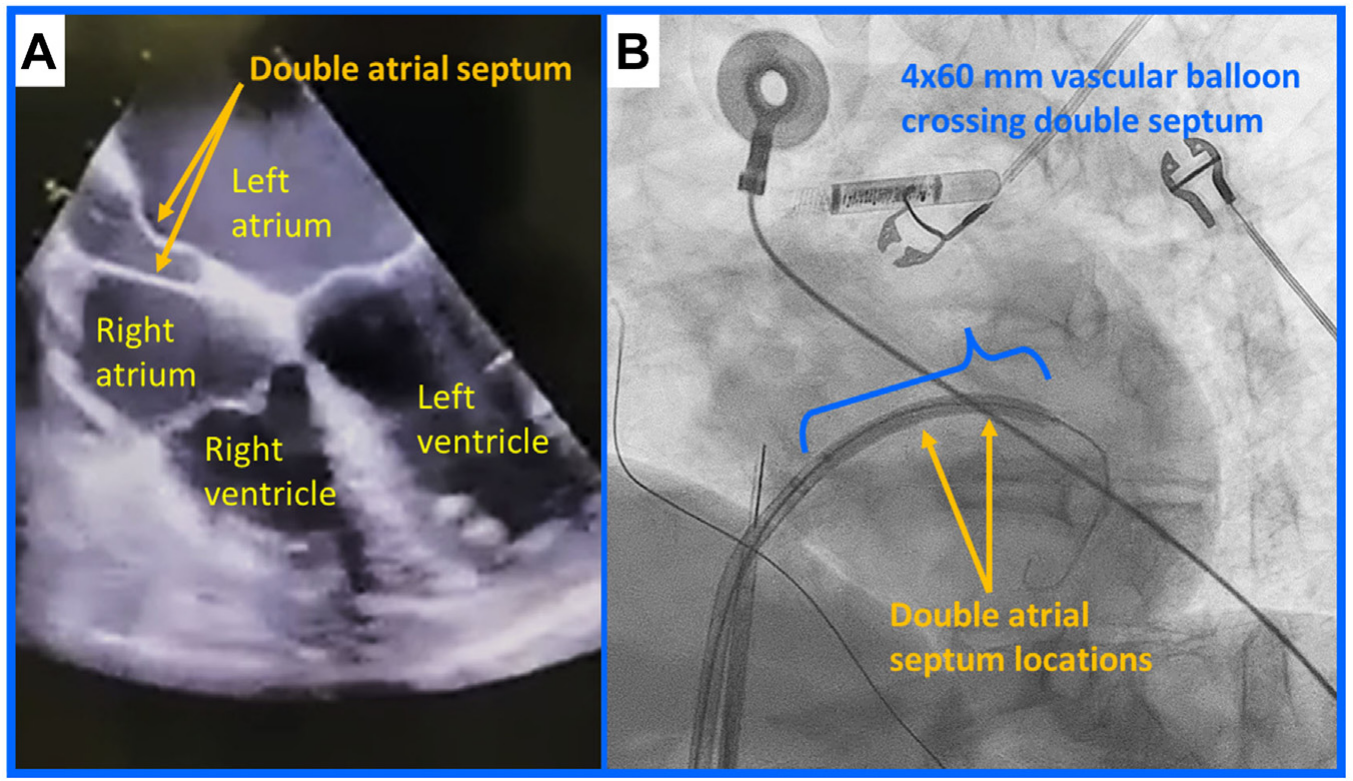
Midesophageal 4-chamber image demonstrating the double interatrial septum. **A:** Note the accessory atrial septum and septum primum separated by an echolucent space. **B:** Armada vascular balloon (Abbott, Diegem, Belgium) deployed and inflated across the double interatrial septum.

A 55-year-old woman with symptomatic paroxysmal AF (including palpitations, fatigue, and dyspnea) presented for PFA treatment. Besides the AF, the patient had a history of hypertension and obesity. A preprocedure transesophageal echocardiogram demonstrated a double interatrial septum (Figure 1A), a rare anatomic anomaly.^3^, ^4^, ^5^ There have been fewer than 20 cases reported in the literature.

The procedure was performed under general anesthesia, and the right groin was prepped and draped using sterile techniques. A 10F sheath (for an intracardiac ultrasound), 7F sheath (for a coronary sinus diagnostic catheter), and Baylis sheath were placed in the right femoral vein. Heparin was administered in a bolus to achieve activated clotting time >350 seconds, and this level of anticoagulation was maintained throughout the ablation procedure via repeated heparin administration. Intracardiac ultrasound (ViewFlex Xtra; St Jude Medical, Irvine, CA) was used throughout the case to monitor transseptal crossing and device navigation. A Baylis sheath and needle (Baylis Medical, Montreal, Canada) were used for transseptal access. The needle entered the left atrium, but the dilator and sheath assembly could not be advanced through the double interatrial septum. Consequently, the Baylis wire was placed into the left atrium (and through the left superior pulmonary vein), and subsequent attempts to manipulate the Baylis sheath into the left atrium were unsuccessful. Specifically, the second septum provided enough restriction that it prohibited the passage of the assembly. The dilator/sheath could not pass through the double-wall septa, and more pushing force only displaced the wire. Also, a decision was made to not use the VersaCross RF wire (Baylis Medical, Montreal, Canada) because of the potential risk that it may loop/coil between the septa and result in a complication.

Ultimately an Armada 4 × 60 mm vascular balloon (Abbott, Diegem, Belgium) was placed over the wire and advanced through the double interatrial septum. The balloon was positioned midway between the right and left atrium. Two inflations were performed, each for 60 seconds at 8 atmospheres (Figure 1B). This allowed the Baylis sheath to subsequently enter the left atrium. The Baylis sheath was then exchanged, albeit with some difficulty, for a 10F FlexCath Contour (Medtronic, Minneapolis, MN). The patient underwent successful pulmonary vein isolation using a multielectrode (25 mm diameter) 9F PFA catheter (PulseSelect; Medtronic, Minneapolis, MN), which delivered a bipolar, biphasic pulsed electric field waveform of 1500 V (amplitude from baseline to peak). Following the exit of the transseptal sheath, intracardiac ultrasound was used to evaluate both septa. There was no evidence of an atrial septal defect at the end of the case, and shunting between atria was not observed. Potentially, the transseptal location (low and anterior)^2^ and the presence of a double-walled septum were conditions that facilitated the prevention of blood shunting between atria. At the end of the procedure, protamine was administered, 2 figure-of-8 sutures were placed, all sheaths were removed, manual pressure was held, and hemostasis was achieved. The patient was discharged per hospital standards of care the next morning, and she continues to be monitored for AF through regular follow-up visits.

## Discussion

There have been several reports in the literature outlining the difficulty associated with transseptal access in patients with a double interatrial septum,^5^, ^6^, ^7^ and yet, there are no reports of techniques or methodology during a PFA catheter usage to support the treatment of patients with AF. To our knowledge, we report the first case of balloon septostomy to facilitate PFA in a patient with a double interatrial septum, and electrophysiologists may find this technique helpful in order to ensure safe transseptal access during difficult cases.

After reviewing the published literature, Karanam and colleagues^8^ described a balloon dilation of a single septum to facilitate entry into the left atrium for left atrial tachycardia ablation. In our case, the placement of the 10F FlexCath Contour sheath into the left atrium was made difficult because of the presence of a second atrial septum that prohibited easy exchange across both septal barriers. However, we may have had a procedural failure if the catheter ablation was dependent upon the older 12F FlexCath sheath used for cryoablation or the 13F sheath required for Farapulse PFA.

In this description, we have demonstrated a method of septal crossing that facilitated entry; however, there were potentially other device-specific advantages that may have facilitated the procedural completion. Mainly, the PulseSelect system offers a bidirectional deflection of both the ablation catheter and sheath, and also, the transit and maneuvering of the system is made easier because of the 9F ablation catheter and 10F sheath. Consequently, in this system a septal approach that is not low and anterior because of prohibitive anatomy can still be completed because of the deflectability within the system and smaller-diameter sheath. In our case, the approach toward the right-sided pulmonary vein ablations via pulsed field was not hampered by the double atrial septum, and the multielectrode PFA was able to easily ablate the circumference of each pulmonary vein, which completed the ablation with confirmed block at each pulmonary vein without any complication.

## Conclusion

In our case, the usage of a vascular balloon facilitated transseptal crossing in a patient with a double atrial septum. A novel multielectrode PFA catheter and system using a small-diameter delivery with dual-deflection maneuverability allowed for successful isolation of all pulmonary veins. To our knowledge, this is the first reporting of this crossing technique used to support a PFA procedure.

## Disclosures

Dr Souza is a consultant for Cardiac Ablation Solutions, Medtronic, Inc, Minneapolis, MN. Dr Lim is an employee of Medtronic, Inc, Minneapolis, MN. Drs Nelson, Reynolds, Dhawan, and Antimisiaris do not have any conflicts regarding this manuscript.
